# Transcranial focused ultrasound to nucleus accumbens reverses fentanyl-induced conditioned place preference in rats

**DOI:** 10.1371/journal.pone.0329748

**Published:** 2025-08-22

**Authors:** Seung-Schik Yoo, Madeline S. Ewell, Evan C. Smith, Isabel L. Yu, Hyun-Chul Kim, Rajeev I. Desai

**Affiliations:** 1 Department of Radiology, Brigham and Women’s Hospital, Harvard Medical School, Boston, Massachusetts, United States of America; 2 Behavioral Biology Program, Integrative Neurochemistry Laboratory, McLean Hospital, Belmont, Massachusetts, United States of America; 3 Center for Drug Discovery, Departments of Chemistry and Chemical Biology and Pharmaceutical Sciences, Northeastern University, Boston, Massachusetts, United States of America; 4 Department of Psychiatry, Harvard Medical School, Boston, Massachusetts, United States of America; University of Colorado Anschutz Medical Campus, UNITED STATES OF AMERICA

## Abstract

Fentanyl-related opioid use disorder (OUD) continues to be a substantial public health concern. Current pharmacological treatments, using methadone and buprenorphine, can cause adverse side effects, and patients often relapse, necessitating alternative treatment strategies. Transcranial focused ultrasound (tFUS), a noninvasive neuromodulation technique with deep brain penetrability and high spatial selectivity, is emerging as a potential intervention for treatment of OUD. To evaluate utility of tFUS in modulating the function of reward circuitry, we applied functionally suppressive tFUS to the nucleus accumbens (nAcc) of male rats to determine if it attenuated fentanyl-induced conditioned place preference (CPP). Fentanyl CPP scores were significantly attenuated following tFUS administration to the nAcc, suggesting tFUS application weakens the rewarding effects of fentanyl in rats. Physiological, behavioral, and neuropathological endpoints were unaffected by tFUS, confirming that other factors did not contribute to the observed effects. Taken together, these results support the use of tFUS as a potential treatment strategy of OUD by non-invasively modulating activity in key reward-related brain regions implicated in OUD.

## Introduction

Fentanyl is widely recognized as a primary contributor to the current epidemic of opioid use disorder (OUD) in the United States, accounting for over 80% of all opioid-related deaths [1–6]. Standard of care medication for OUD utilizes oral methadone or buprenorphine (mu-opioid receptor [MOR] full/partial agonists) to reduce opioid withdrawal symptoms and maintain abstinence [7–11]. However, these treatments can cause some adverse side effects, often leading to loss of patient compliance [12–15]. Also, OUD medications show low abstinence rates–about 66% of patients will relapse in 6 months post-treatment [7,16–19]. For acute fentanyl overdose, the MOR antagonist naloxone is currently the only approved and highly effective treatment. However, due to the increased potency of fentanyl, coupled with a short duration of action, multiple doses of naloxone are required [20–22]. In general, MOR antagonists, including naloxone, may precipitate withdrawal and block endogenous opioids, which leads to a dysphoric state in patients [12,14]. Although the existing MOR treatments for OUD and acute fentanyl overdose demonstrate success in most patients, the high recidivism and increasing overdose rate calls for an urgent need for alternative treatment strategies aimed at attenuating fentanyl-related OUD .

A growing body of evidence indicates that dysfunction of dopamine (DA) activity within the nucleus accumbens (nAcc), one of major components in reward-related DA circuitry, is a common denominator of the abuse-related neurochemical and behavioral effects of most psychoactive drugs, including opioids like fentanyl [23–29]. However, pharmacotherapies targeting DA mechanisms to manage the widespread use of substance use disorders (SUD) has shown limited success [30–34]. This may be because neurochemical systems in addition to DA (e.g., glutamate and γ-aminobutyric acid [GABA]) also appear to contribute to the addiction-related effects of opioids [35–38]. In addition, it is well-established that endogenous opioid receptors and opioid systems play a critical role in mediating the abuse-related effects of opioids–and particularly in reward-related brain regions such as the nAcc [35–38]. Thus, while most studies have separately documented the contribution of individual neurotransmitters in the abuse-related behavioral actions of opioids and other psychoactive drugs [35–38], a comprehensive analysis of DA dynamics relative to changes in other neurochemicals in the nAcc shell or other reward-related brain regions (e.g., ventral tegmental area) has not been systematically documented.

Given the above, an alternative, and perhaps more attractive approach for treating SUD may lie in the use of non-pharmacological, noninvasive brain stimulation (NIBS) that targets regions known to be involved in reward circuitry. Existing NIBS techniques, such as transcranial magnetic stimulation (TMS) and transcranial direct/alternating current stimulation (tDCS/tACS), offer non-pharmacological alternatives to invasive procedures such as deep brain stimulation and vagus nerve stimulation [39–42]. Recent studies have begun to explore the potential of deep brain stimulation (DBS) as a therapeutic intervention for treatment-resistant OUD [43,44]. One prospective clinical trial targeting the nAcc and ventral capsule demonstrated that DBS can be safely administered in individuals with severe OUD, with some participants achieving meaningful and sustained reductions in drug use and associated symptoms such as craving and depression [45]. Such observations highlight broader therapeutic effects across different SUDs, pointing to DBS as a promising neuromodulatory approach for addiction. However, a technique that selectively targets the nAcc to modulate activity such that the abuse-related behavioral effects of psychoactive substances like fentanyl are attenuated has not been developed. While studies have reported that TMS and tDCS/tACS may alter cortical activity affecting brain reward and reduce addiction cravings, they cannot reach deep brain areas with sufficient spatial selectivity [46,47]. Given the increase in fentanyl-related OUD, combined with the limitations of current treatment options, novel, safe, and effective non-invasive strategies aimed at suppressing rewarding activity in the nAcc are urgently needed for treating fentanyl-related OUD.

Transcranial Focused ultrasound (tFUS), a noninvasive technology with deep brain penetrability and high spatial selectivity [7,48–50], is emerging as a powerful approach to address this need by modulating OUD-related neurobiological activity in targeted brain regions. FUS sonication, given in a batch of pulses at a low intensity below the threshold for heat generation or mechanical damage, can reversibly modulate (i.e., either increase or suppress) the excitability of regional brain tissue [48,51–53]. tFUS has gained momentum as a new NIBS technique due to its exquisite spatial selectivity and depth control over existing modalities, along with a promising safety record in both small (e.g., rodents) and large (e.g., sheep) animals [48,53–56], nonhuman primates [57,58], and humans [50,59–62]. In addition, emerging evidence indicates that the modulatory effects of FUS outlast the duration of sonication [50,58,63,64], which is critical for its therapeutic effects to occur [65,66]. Indeed, recent first-in-human studies reported that application of tFUS to the nAcc drastically reduced caving ratings for a variety of drugs of abuse including benzodiazepine, cannabis, alcohol, and nicotine, for up to 3 months without nonspecific unwanted effects [8,9]. Such observations demonstrate that tFUS ought to be developed as a viable NIBS alternative for treating SUDs.

In the present study, we were motivated to examine whether tFUS-mediated suppression of nAcc in rodents, using the sonication parameter that has previously been shown to suppress the excitability of brain tissue in rodents and large animals (e.g., sheep) [48,53–56], will modify the rewarding effects of fentanyl in the conditioned place preference (CPP) assay. To probe the translational potentials of tFUS for out-patient settings, we utilized a single-element FUS transducer configuration in which stereotactic image-guidance has shown to effectively engage the brain targets in humans without the need for the surgical-grade preparations [50,55,60,62,67]. Furthermore, the safety of the tFUS was assessed by conducting a comprehensive histological analysis of the sonicated brain tissue across different time points, spanning acute (within 1 hr), intermediate (1 day and 1 week), and long-term (4 weeks) timepoints after the tFUS application.

## Materials and methods

### Materials

#### Subjects.

Eight experimentally-naïve adult male Sprague-Dawley rats (Charles River Laboratories, Inc., Wilmington MA) weighing 238.6 ± 90.1 g were used. All rats were group-housed (n = 2) in a temperature- and humidity-controlled colony with a 12-h light/dark cycle (lights on at 7:00 a.m.). CPP experiments were conducted during the light phase between 10:00 a.m. and 1:00 p.m. All rats were maintained at 7.0 ± 2.3% (max 10%) of the free feeding weight and were fed the standard laboratory chow (Rodent Diet 20, LabDiet, St. Louis, MO). Water was freely available except during experimental sessions. This study was carried out in strict accordance with the recommendations in the Guide for Care and Use of Laboratory Animals of the National Institutes of Health (National Academy of Sciences, 2011) [68]. All animal procedures and protocols (Protocol #: 2023N000060) were conducted according to the regulations and standards of Brigham and Women’s Hospital Institutional Animal Care and Use Committee (IACUC), and the study was carried out and reported in accordance with the ARRIVE guidelines (https://arriveguidelines.org/). All tFUS procedures were performed under isoflurane anesthesia (3% for induction and ~1.5% for the maintenance; see below for further details), and all efforts were made to minimize suffering.

#### Apparatus.

The CPP experiments were conducted in acrylic chambers that consisted of two equally sized (30 × 30 × 30 cm, L × W × H) compartments, one white and the other black. The white and black chambers were lined with a meshed black plastic mat (33 mm square grids) and line-gridded white mat (6 mm spacing), respectively. The two chambers were interconnected by a gray chamber (9 × 14 × 30 cm, L × W × H) and sliding doors (9 × 14 cm, W × H) that opened vertically, with its matching color facing each side of the chamber. The gray chamber had a transparent plastic mat with 6 mm holes spaced at 12 mm. The white and black chambers had white lighting (~9.5 Lux; MT-912, Urceri, Shenzhen, China) and the middle grey chamber had slightly brighter lighting conditions (~18 Lux). The time spent in each compartment was recorded and generated by a video camera placed over the CPP chamber (Logitech, Lausanne, Switzerland). The raters were blinded to the sequence of experiment during video analysis.

#### tFUS device and operation.

**Fig 1:**
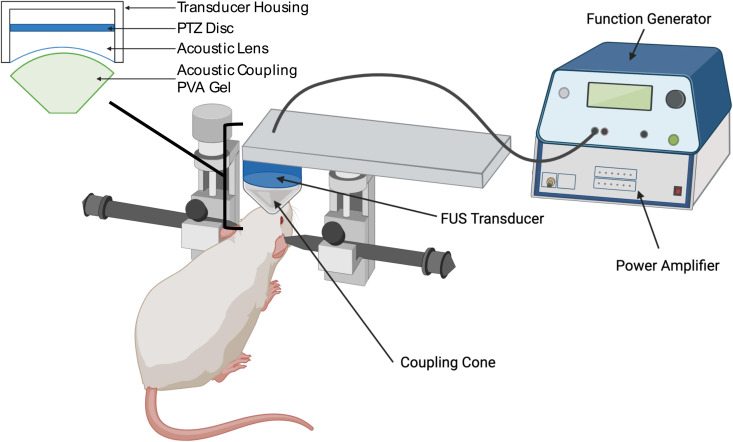
A schematic of the tFUS device and operation. The ultrasound, generated by a lead zirconate titanate (PZT) ceramic disk with a diameter of 19.1 mm (APC International Ltd., Mackeyville, PA), was focused by a plano-concave (20 mm radius-of-curvature) polyetherimide acoustic lens abutted to the disc (Armset LLC, Middleton, MA). The transducer was actuated with pulsed sinusoidal waves generated from a function generator (33210A, Keysight, Santa Rosa, CA), and the spatial profile of the acoustic field was separately mapped in degassed water using a needle-type hydrophone (HCN, Onda Corporation) mounted to a 3-axis mechanical stage (BiSlide, Velmex Inc., Ontario County, NY), using methods previously described (Yoo et al., 2011) [48] .

**Fig 2:**
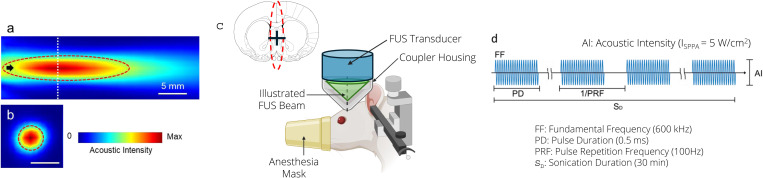
Spatial profile of acoustic intensity (W/cm^2^) from the transducer. Profile covers a 31 × 31 mm^2^ transversal plane and a 51 × 31 mm^2^ longitudinal plane along the sonication axis. The intensity map on the longitudinal plane (a) was measured 10 mm away from the exit plane of the transducer while the transverse profile (b) was measured at the acoustic focus (located 21 mm away from the exit plane), indicated as a white dotted line. The FWHM intensity profile is depicted with dotted red lines. The black arrow indicates the direction of sonication. (c) FUS setup for a rat. The green inverted triangle depicts the sonication to the nAcc, the major terminal area of the mesolimbic DA system. The red dotted lines and the black cross illustrate the sonication path targeting the nAcc (Paxinos and Watson, 1987) [69]. (d) The illustration of the acoustic parameters used.

A single-element FUS operating at fundamental frequency of 600 kHz (26.5 mm in diameter and 26.5 mm in height, weighing 19 g) was made in-house. Fig 1 shows a schematic of the tFUS device and operation. The ultrasound, generated by a lead zirconate titanate (PZT) ceramic disk with a diameter of 19.1 mm (APC International Ltd., Mackeyville, PA), was focused by a plano-concave (20 mm radius-of-curvature) polyetherimide acoustic lens abutted to the disc (Armset LLC, Middleton, MA). The transducer was actuated with pulsed sinusoidal waves generated from a function generator (33210A, Keysight, Santa Rosa, CA), and the spatial profile of the acoustic field was mapped in degassed water using a needle-type hydrophone (HCN, Onda Corporation) mounted to a 3-axis mechanical stage (BiSlide, Velmex Inc., Ontario County, NY), using methods previously described [48]. The acoustic focus was formed 21 mm away from the exit plane of the transducer. The size of the focus was 5 mm in diameter and 22 mm in length, at the full-width at half-maximum (FWHM) of the intensity profile (Figs 2a and b).

The sonication parameter targeting the nAcc (Fig 2c) was chosen based on previous studies that suppress the excitability of brain tissue in rodents and sheep, where 0.5 ms pulse duration (PD) using 100 Hz pulse repetition frequency (PRF) were used at 5 W/cm^2^ spatial-peak pulse average intensity (I_SPPA_) (Fig 2d) [53,54,56]. The parameter has been extensively studied in terms of its non-thermal/non-destructive nature. The corresponding peak rarefactional pressure (P_r_) was 0.38 MPa, yielding a mechanical index (MI) of 0.49 (P_r_ [in MPa]/√Frequency [in MHz]; Fig 2d), much lower than the safety limit on MI of 1.9 for soft tissue ultrasound imaging set by the U.S. Food and Drug Administration (FDA). Considering 5% duty cycle of the sonication, spatial-peak temporal-average intensities (I_SPTA_) at the focus was 250 mW/cm^2^, much lower than the upper limit of 720 mW/cm^2^ I_SPTA_ for diagnostic ultrasound imagers set by the FDA [70].

#### Drugs.

Fentanyl citrate was obtained from Hikma Pharmaceuticals (Eatontown, NJ). Fentanyl was dissolved in 0.9% saline. 17 µg/kg fentanyl was administered subcutaneously (s.c.) in a volume of 1.0 ml/kg, and the dose was expressed in terms of salt.

### Methods

#### CPP studies – preconditioning phase.

**Fig 3:**
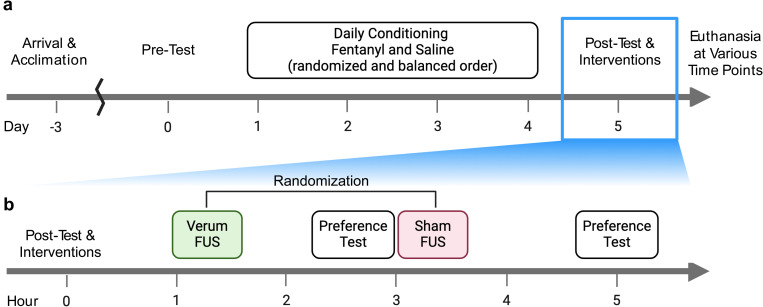
Schematics of experimental procedures. (a) Upon acclimation of the animals, initial place preference was evaluated on Day 0 (‘Pre-Test’). Starting Day 1, fentanyl and saline were subcutaneously injected in a randomized and balanced fashion, producing place conditioning. The process repeated daily for three additional days. On Day 5, the animal was evaluated for the place preference (‘Post-Test’), followed by verum/sham FUS interventions and evaluation of their preference following the interventions. Then, the animals underwent different waiting periods until sacrifice. (b) Detailed experimental procedures on Day 5. The animal underwent either verum or sham FUS session (sequence randomized and balanced across the animals) after the ‘Post-test’. ~ 2hr gap was introduced between the initiation of verum/sham FUS to allow for recovery from anesthesia.

Fig 3 shows a timeline of the experimental protocol used to evaluate the effects of tFUS on fentanyl’s rewarding effects. Each animal was acclimated to the test chambers (1 ~ 1.5 hrs) for 3 successive days prior to the initial pre-conditioning phase (‘Pre-Test’; Fig 3a). On the day of Pre-test (day 0), the animal was placed in the middle grey chamber and allowed to have uninterrupted access to both chambers for 20 min. The time spent by each animal in each chamber was recorded. Exclusion criteria for strong initial preference by individual subjects for either the black or white compartment was set to > 30% to ensure an unbiased CPP design, i.e., the subject cohort was without inherent predisposition for preference of a particular compartment. In the present study, no rats displayed such initial preference, and therefore none were excluded from the study.

#### CPP studies – fentanyl-induced CPP.

During the conditioning phase, each animal was administered with either fentanyl or saline prior to two 30-min daily conditioning sessions in either the white or black compartments at ~10:00 AM and ~2:00 PM (Fig 3a). Immediately after injection, rats were placed individually in one compartment with access to the other compartment blocked. The black and white compartments were randomly assigned with drug or saline, and the sequence of conditioning was randomized and counterbalanced across animals. These conditioning sessions were repeated for three additional days (total 4 days; Fig 3a). After each conditioning session, rats were immediately returned to their home cage. On day 5, ~ 24 hr after the last conditioning session, the effects from the conditioning were evaluated, being followed by verum and sham tFUS interventions (‘Post-Test & Interventions’, Fig 3b).

#### CPP studies – effects of tFUS on fentanyl CPP.

On day 5, the fentanyl-induced CPP was first evaluated. Next, FUS or sham FUS were transcranially delivered to the nAcc for 30 min (noted as ‘verum’ and ‘sham’ respectively; Fig 3b). Briefly, under isoflurane anesthesia (3% for induction and ~1.5% for the maintenance), the fur over each rat’s head was removed to provide an uninterrupted path for FUS delivery to the nAcc. Each rat was placed onto a 3-axis robotic platform and demobilized using an ear and bite bar. FUS was stereotactically delivered to the nAcc (1 mm rostral to the bregma and 7 mm in depth, as reference to interaural line). The sham condition had an identical tFUS procedure, including anesthesia and placement of the FUS transducer coupled to the scalp, however, the animal received zero acoustic output from the device. The respiratory/heart rates and oxygen saturation (SpO_2_) were measured (V3402, Surgivet, Waukesha, WI) before, 15 minutes after, and completion of sonication (either verum or sham) duration. To couple the transducer surface to the skull, cone-shaped compressible polyvinyl alcohol (PVA) hydrogel (7%, 2 freeze-thaw cycles) was used, and ultrasound gel (Aquasonic 100, Parker Lab, Fairfield, NJ) applied to all interfaces. The sequence of verum and sham tFUS intervention was randomized and balanced, each followed by post-intervention CPP evaluation that occurred ~1-hr after the full recovery from the anesthesia. Each verum/sham tFUS intervention started with 2-hour gap in-between (Fig 3b).

#### Histological analysis.

After completing the experimental procedure, the animals were divided into 4 groups to examine the acute (within 1 hr, n = 2), intermediate (1 day and 1 week, n = 2 each) and long-term (4 weeks, n = 2) biological effects after FUS delivery. For brain extraction, animals were deeply anesthetized with ketamine/Xylazine (80 mg/kg ketamine and 10 mg/kg Xylazine) and underwent transcardial perfusion using normal saline and 10% formalin solution (SF100–20, Thermo Fisher Scientific). Then brains from all subjects were extracted after an additional 24 hr of immersion fixation in 10% formalin. The extracted brain was sectioned on the sonicated plane (rostral/caudal directions) and underwent histological analysis examining the presence of necrosis or neuronal loss, ischemic neurons, glial infiltration and degenerated neurons, or apoptosis through hematoxylin and eosin (H&E), vanadium acid fuchsin (VAF)-toluidine blue, glial fibrillary acidic protein (GFAP) immunohistochemistry (IHC), and caspase-3 IHC, respectively [71–73].

### Data analysis

All data analysis was conducted in GraphPad Prism 10.

#### Vital signs.

The respiratory/heart rates and oxygen saturation levels during anesthesia for the intervention are reported as an average ±SEM, with all subjects displayed as one mean. The group means were analyzed with multiple paired t-tests. Effects where p < 0.05 were considered statistically significant.

#### CPP scoring.

The time spent in chambers was measured using video analysis software (MPC-HC, ver. 2.3.9, in 10 ms time resolution). The raters were blinded to the sequence of experiments during video analysis. Analysis of CPP data divided time into two categories: paired chamber time, time allocated to either the saline or fentanyl paired chambers, and unpaired time, time allocated to the starting middle chamber. CPP score, a measure of the preference produced by the conditioning phase, was determined by subtracting the time spent in the saline paired chamber from the time spent in the fentanyl paired chamber. Both group-level fentanyl CPP score, and the time spent in the starting middle chamber were shown as average time spent in the chamber in seconds ±SEM. The effects of tFUS treatments were further analyzed by subtracting fentanyl-induced CPP score (on Day 5) from either sham or verum CPP score to represent treatment effects (TE_sham_ or TE_verum_, respectively). These were expressed as average ±SEM, with each animal individually displayed as paired results for each treatment. The group means were analyzed by one-way repeated measures ANOVAs followed by paired t-tests of treatment versus verum and sham controls to determine treatment effects. Paired *t* tests were also used to compare TE_sham_ or TE_verum_ treatment effects. Effects with determined *P* < 0.05 were considered statistically significant.

#### Motor activity.

For each rat, the time spent conducting “motor” behaviors in each of the three chambers was recorded to determine the total motor activity across the duration of the session. The categories of four motor behaviors are based on our prior work [73,74] and included: a) ambulation: horizontal displacement of the body in the space; forward walking, darting movements or running, b) rearing: rearing body in vertical or near-vertical plane with or without front paws against the wall, c) sniffing: sniffing chamber floor, walls, air or any other form of sniffing, and d) grooming: face and body washing strokes with the forepaws, scratching with hindlimbs, anogenital licking and tail nibbling. Behaviors were scored using the open-source Behavioral Observation Research Interactive Software (BORIS) and grouped to keep a record of standard activity to assess whether both fentanyl and the sonication parameter interfere with the animals’ overall motor activity. The motor activity is reported as an average ±SEM. The group means were analyzed by a two-way ANOVA. Effects where p < 0.05 were considered statistically significant.

## Results

### Vital signs

**Fig 4:**
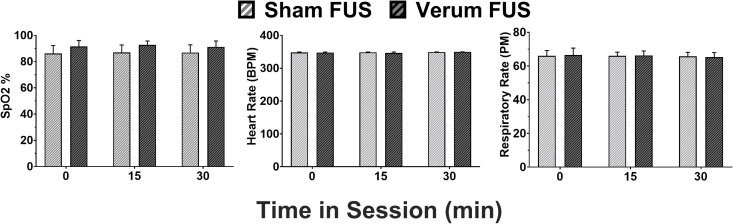
Effects of sham and verum FUS administration on (a) SpO2, (b) heart rate (beats per minute), and (c) respiratory rate before (0 min), during (15 min), and after (30 min) both treatment in rats. Each data point represents all subjects tested at each treatment condition and time point (n = 8).

Heart rate, respiratory rate, and SpO_2_ were recorded before, during, and after both sham and verum tFUS conditions during anesthesia (Fig 4). Results show that the vital signs of SpO_2_, heart rate, and respiratory rate were not different before (SpO_2_: 91.50% ± 4.57 vs 86.13% ± 6.16; heart rate: 347.4 ± 1.84 vs 348.1 ± 1.32 beats/min; respiratory rate: 66.50 ± 4.14 vs 66 ± 3.23 pulse/min; verum and sham, respectively; **ts* *= 0.37 to 1.43; *Ps* ≥ 0.197), during (SpO_2_: 92.75% ± 2.99 vs 87% ± 5.71; heart rate: 346.5 ± 2.97 vs 348.7 ± 1.10 beats/min; respiratory rate: 66.25 ± 2.66 vs 66 ± 2.24 pulse/min; verum and sham, respectively; **ts* *= 0.20 to 1.18; *Ps* ≥ 0.275), and after (SpO_2_: 91.25% ± 4.48 vs 86.88% ± 5.92; heart rate: 349.9 ± 0.13 vs 349.3 ± 0.53 beats/min; respiratory rate: 65.25 ± 2.75 vs 65.75 ± 2.37 pulse/min; verum and sham, respectively; **ts* *= 0.80 to 1.26; *Ps* ≥ 0.25) the sham and verum FUS procedures.

### tFUS-mediated attenuation of fentanyl CPP

**Fig 5:**
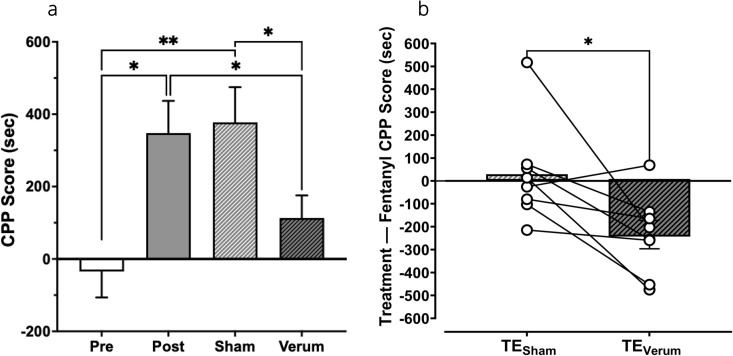
Attenuation of 17 µg/kg fentanyl CPP after the application of verum tFUS to the nAcc. (a) Mean CPP scores (fentanyl time – saline time, in sec) in the pre (before training), post (after training), sham (simulated FUS procedure), and verum (application of tFUS to the nAcc) conditions for rats administered (n = 8). (b) Mean treatment effect (difference between CPP scores before (post condition) and after (sham or verum condition) treatment) on CPP scores (n = 8). *P < 0.05, ** *P* < 0.01.

The impact of tFUS on fentanyl’s rewarding properties was explored using the CPP assay (Fig 5). ANOVA showed that CPP scores were significantly different across treatment groups (F_3,21_ = 10.63, *P* = 0.0009; Fig 5a). Post-hoc analysis confirmed that 4-day administration of fentanyl increased the time difference between the fentanyl- and saline-paired chamber from approximately –36 ± 72s during initial pre-conditioning to approximately 376 ± 89s and 378 ± 97s after the conditioning (*t = *–3.90*, P* = 0.006) and sham treatment (*t* = –5.47, P < 0.001; Fig 5a), respectively. In contrast, after application of verum tFUS to the nAcc, the time difference between the fentanyl- and saline-paired chamber did not significantly differ from the initial pre-conditioning values (~113 ± 62 vs –36 ± 72s; *t* = –1.48 *P* = 0.182; Fig 5a). In addition, verum tFUS significantly reduced the CPP score when compared to the one measured at post conditioning (*t* = 3.79, *P* = 0.007) and after sham tFUS (*t* = 2.85, *P* = 0.025). Fig 5b compares the difference in CPP score (i.e., fentanyl–saline) after verum or sham tFUS with respect to the post conditioning CPP score (represented as TE_verum_ or TE_sham_, respectively) and shows significantly reduced time differentials in the CPP score after verum tFUS compared to the sham condition (t = 2.85, *P* = 0.025).

**Fig 6:**
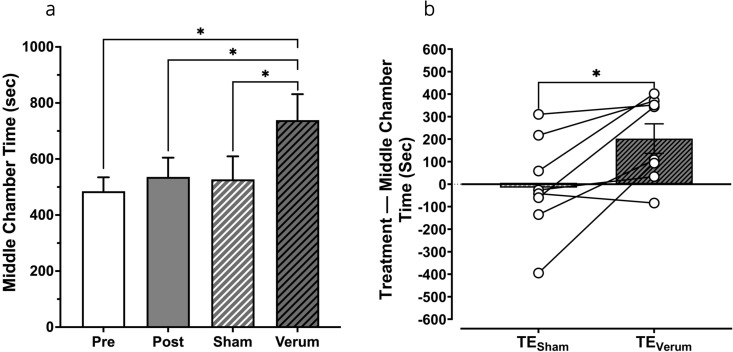
Time spent in the middle chamber across treatment conditions. (a) Mean time spent in the middle chamber across treatment conditions (n = 8). (b) Mean treatment effect (difference between time spent in the middle chamber before (post condition) and the time after (sham or verum condition) treatment, n = 8). *P < 0.05.

The time duration that each animal spent in the middle gray chamber compared to the other two chambers show that the animals spent comparable time in the middle chamber before and after place preference conditioning (~485 ± 50s and ~536 ± 68s, respectively; Fig 6a). This value did not substantially change after the sham tFUS condition (~527 ± 82s, *Ps* > 0.05). However, after verum tFUS, animals spent significantly more time in the middle gray chamber (~738 ± 92s) compared to the pre, post, and sham tFUS conditions (*ts = *–2.41, –3.08, and –3.15; *Ps* = 0.047, 0.018, and 0.016, respectively). Fig 6b compares the differences in the middle chamber time after verum or sham tFUS, with respect to the time spent in middle chamber after post conditioning (‘Post’, Fig 6a). Results show a significantly greater time differential in the middle gray chamber after verum tFUS compared to the sham condition (*P* = 0.016).

### Effects of tFUS on motor activity

**Fig 7:**
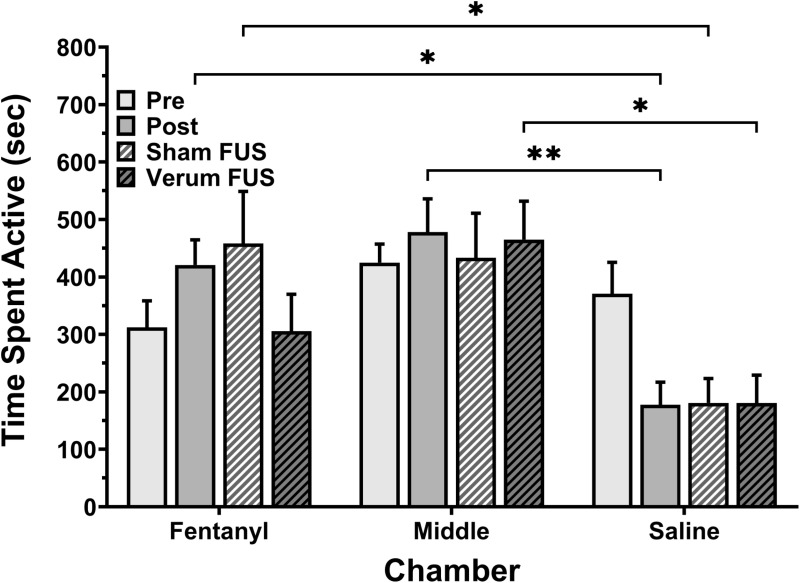
Effects on overall motor activity in each chamber across all four treatment conditions (n = 8). Ordinates: time spent active in seconds. Abscissae: drug, middle, or saline chamber. *P < 0.05, ** *P* < 0.01.

Motor behavior was also evaluated in each of the three chambers throughout the CPP testing to determine whether the effects of tFUS application on fentanyl-induced CPP are behaviorally specific (Fig 7). ANOVA indicated a significant main effect of chamber (fentanyl vs middle vs saline; F_2,56 _= 11.82; *P* = 0.0003). However, a significant effect of condition (pre- vs post- vs sham tFUS vs verum tFUS; F_3,56 _= 1.24; *P* > 0.05) and a chamber/condition interaction (F_6,56 _= 1.62; *P* > 0.05) were not observed (Fig 7). Post-hoc analysis revealed that the significant main effects of the chamber were related to a difference in activity between the saline/fentanyl chambers, saline/middle chambers in the post condition (*Ps* < 0.05), fentanyl/saline chamber in the sham FUS condition (*P* = 0.046), and the middle/saline chamber during the verum FUS condition (*P* = 0.036). Although motor activity decreased slightly in the fentanyl chamber after tFUS administration (305.68 ± 64.00 s) when compared to post conditioning (420.72 ± 43.91 s) and to sham FUS conditions (458.08 ± 90.84 s), these changes were not statistically significant (all *Ps* > 0.05).

### Histological analysis

**Fig 8:**
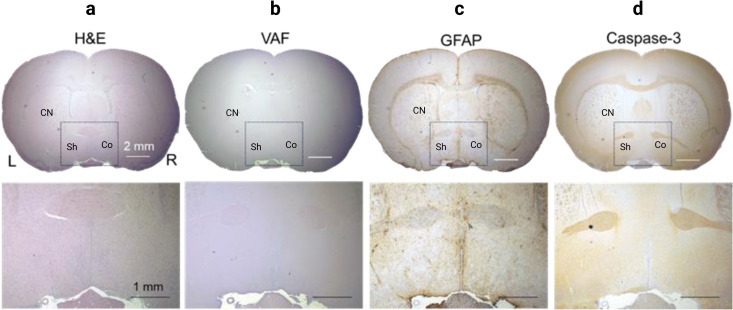
Examples of histological analysis. (From left to right) H&E, VAF, GFAP, and Caspase-3 staining. Top row (x1) and bottom two (x4) from the dotted area of sonication. CN: Caudate Nuclei, Sh: Shell of nAcc, Co: Core of nAcc.

Fig 8 shows representative sections of the sonicated brain. Histological analysis of the sonicated brain regions spanning acute (within 1 hr), intermediate- (1 day and 1 week), and long-term (4 weeks) after the tFUS application did not reveal any major signs of tissue damage in any of the rats tested as indicated by an absence of hemorrhaging (from H&E, Fig 8a), ischemic (VAF-toluidine blue; Fig 8b) and glial infiltrations (GFAP; Fig 8c), including apoptotic damage (caspase-3; Fig 8d).

## Discussion

Neuromodulatory techniques have entered the forefront of research on noninvasive interventions for various diseases, including SUDs. Opioid use – fentanyl in particular – is of growing interest for noninvasive treatment methods. Here, we evaluated how targeting the nAcc with the tFUS neuromodulatory technique impacts fentanyl’s rewarding properties. Remarkably, we found that sonicating the nAcc in rats significantly attenuated fentanyl’s rewarding effects in the CPP assay. After verum tFUS administration to the nAcc, rats spent considerably less time in fentanyl-paired chambers when compared to post-conditioning and sham tFUS condition. Taken together, these data provide strong evidence that targeted tFUS application to the nAcc, via its functional suppression, can successfully modulate reward-related brain regions to attenuate the abuse-related rewarding effects of fentanyl.

Although the sonication parameter set used in this study was previously established to effectively suppress regional cortical and thalamic brain activity [53,54,56], it is possible that other non-specific physiological, behavioral, and/or neuropathological factors during or after sonication of the nAcc also may have contributed to the attenuation of fentanyl-induced CPP. However, several observations in the present study indicate that this is unlikely. For example, we found no major differences in key vital signs of SpO_2_, heart rate, and respiratory rate during anesthesia and delivery of the sham/verum tFUS, which indicate equivalent depth of anesthesia. In addition, analysis of the place preference data revealed an increase in the time spent in the middle chamber after both sham and verum tFUS, suggesting that the application of tFUS to the nAcc does not cause aversive effects that could account for the attenuation of fentanyl’s rewarding properties. Moreover, assessment of general motor activity during the CPP assay revealed no significant differences across experimental conditions, indicating that motor activity levels remained consistent regardless of whether an animal received a tFUS treatment, further supporting our view that tFUS selectively attenuates the rewarding properties of fentanyl without affecting motor activity that could have interfered with its ability to attenuate fentanyl CPP. Assessing the impact of tFUS delivery to the nAcc on non-drug rewards (e.g., food reinforced behavior) will further demonstrate that ultrasound application leads to behaviorally specific disruption of the nAcc to impact fentanyl-induced CPP.

The use of low MI of 0.49 and I_SPTA_ of 250 mW/cm^2^ at the focus was far below the level that can induce thermal or cavitational damage to biological tissue. Along with long track records of safely modulating the regional brain function across different species, including humans, the absence of any abnormal structural and cellular histological feature in the sonicated brain area at different time points, spanning acute (within 1 hr), intermediate- (1 day and 1 week), and long-term (4 weeks) after the tFUS application corroborates the safety of this technique. Taken together, these data further support our view that other physiological, behavioral, or pathological factors did not contribute to tFUS’s ability to attenuate fentanyl’s rewarding effects in the CPP assay.

At present, the underlying mechanisms responsible for the attenuation of fentanyl-induced CPP remain unclear. Given that nAcc opioid, DA, glutamate, and GABA activity has been shown to play a major role in the abuse-related behavioral effects of opioids like fentanyl and most other psychoactive drugs [23–29], we conjecture that the attenuation of fentanyl reward is related to FUS-mediated modulation of this complex neurotransmitter dynamics in this brain region. However, further studies probing the effects of tFUS on neurochemical activities are necessary to confirm this view and elucidate the underlying mechanisms responsible for the disruption of CPP. As the responses to the ultrasound may vary depending on cell type [75–77], research on determining the optimal sonication parameters for maximally modulating neurotransmitter function in the nAcc is warranted. Utilization of *in vitro* cell culture platform to measure excitability of these cells or direct measurement of the DA and other neurotransmitter levels modulated by the sonication (e.g., via *in vivo* microdialysis) may help to elucidate the mechanisms underlying ultrasound’s effects. Despite being speculative in the absence of additional data, the attenuation of fentanyl reward and the consequent increase in time spent in the middle chamber could be related to the effects of tFUS on normalizing fentanyl-induced changes in neurochemical activity the nAcc, which in turn returns fentanyl-induced CPP to baseline levels.

Notably, recent in-human studies reported that tFUS application to the nAcc can block the effects of a wide range of psychoactive drugs, including opioids [8,9]. While these clinical outcomes are highly encouraging, it is unclear whether the observed effects stemmed from either excitation or suppression of the nAcc activity. This is especially germane as the choice of sonication parameter is known to induce differential modulation of the brain tissue excitability [12,13,20,53]. Moreover, the multi-array FUS transducer configuration used in human demonstration of the tFUS for the treatment of SUD involves surgical-grade preparations for accurate target engagement (e.g., administration of head-fixation frame and hair shaving) [78], which often involve more than temporary discomfort to the individuals, thus limiting its wide-spread clinical utility.

Clearly, further research is required to better understand how tFUS may be applied in clinical settings, and the mechanisms by which reward circuitry is being modulated. In this regard, preclinical studies in laboratory animals may provide critical information that may guide the further development of tFUS technology as a viable NIBS alternative for treating SUDs. Despite the established disruption of fentanyl-induced CPP, it is possible that this attenuation is relatively short-term and that the effects are more related to disruptions on memory processes associated with CPP rather than modulation of the reward circuitry involved in fentanyl’s abuse-related effects. It is possible that several aspects of the experimental approach, i.e., wash out period between conditions, order effects, may have interfered with the observed effects. In this regard, although it was not done in the present study, our previous studies on stimulating somatosensory areas in rats had shown changes in somatosensory evoked potential returns to baseline level in about 40 min [63]. Furthermore, others have shown that behavioral modulation (e.g., motor evoked response) typically resolves within minutes in rodents, providing a justification for 2-hr wash-out period between conditions in the present study [79,80]. Also, since the sequence of verum and sham FUS sessions were randomized and balanced, if there were significant ‘spill over’ effects between the conditions, the effects from one condition might have averaged out. Nonetheless, further studies will reveal the lasting effects and duration of FUS, which requires the separation of two FUS conditions in different groups of animals.

It is noteworthy that studies conducted in animal models and humans have shown emerging evidence that the functional modulatory effects of tFUS last for at least 30 minutes beyond the duration of the sonication (see references below in rodents, primates, and humans), highlighting the possibility of inducing the neural plasticity needed for therapeutic intervention [50,58,63,64,81,82]. However, as neuronal responses will be cell-type specific and parameter-dependent, further studies are needed to assess the duration of FUS effects, as well as frequency of treatments that can induce desired modification of neurotransmitter activities through neural plasticity. Moreover, it is possible that other off target effects, including the potential for induction of anhedonia could have contributed to the greater time spent in the middle chamber following tFUS. Clearly, additional studies are needed to systematically address these and other issues that will further strengthen our findings and render our results more generalized for easier translation to humans. For example, the use of a between-subjects design in future investigations will help better understand the potential short- and long-term impact of tFUS application on fentanyl’s abuse-related effects. Additionally, evaluating the effects of tFUS delivery to other opioid reward-related (e.g., prefrontal cortex and ventral tegmental area) and non-opioid reward-related (e.g., hippocampus, motor cortex) brain regions will provide further information on how modulating activity in different brain regions of the reward circuitry impacts fentanyl’s abuse-related effects. We note that the focal dimension created by a single-element FUS transducer may elicit modulation of brain regions other than intended nAcc (e.g., the septum). Future studies may consider adopting higher frequency to create a sharper focal area or implementing acoustic hologram that can generate multiple focal regions using implementation of an acoustic lens through time-reversal technique [83]. Another limitation of the studies reported here is that the effects of tFUS were determined in male rats only. Future studies in both male and female rats will elucidate any sex differences in animal response to tFUS. Ultimately, these data will advance our understanding of how neuromodulatory techniques may be used to treat fentanyl-related OUD and other SUDs.

Our preclinical data in rodents have established that suppressive tFUS sonication parameters may be effective for modulating reward-related brain regions to selectively attenuate fentanyl’s addiction-related effects. Moreover, unlike the human studies described above, here we developed and utilized a single-element FUS transducer which does not require surgical-grade preparations for accurate target engagement which permits repeated application of tFUS in an outpatient clinical setting – considerations that likely improve patient compliance. Use of single-element transducers have been widely adopted in functional neuromodulation studies [84,85], as well as non-invasive applications for treatment of several disorders, including depression, Alzheimer’s Disease, and epilepsy [86–88]. The successes of these studies further support the use of a single-element transducer and tFUS application to treat OUD.

## Conclusion

Taken together, the present results demonstrate that tFUS selectively attenuates fentanyl’s reward-related effects and therefore ought to be further developed as a viable non-invasive, and non-pharmacological NIBS alternative treatment strategy for treating fentanyl-related OUD and other SUDs.
